# Longitudinal modeling of ultrasensitive and traditional prostate-specific antigen and prediction of biochemical recurrence after radical prostatectomy

**DOI:** 10.1038/srep36161

**Published:** 2016-11-02

**Authors:** Teemu D. Laajala, Heikki Seikkula, Fatemeh Seyednasrollah sadat, Tuomas Mirtti, Peter J. Boström, Laura L. Elo

**Affiliations:** 1 Computational Biomedicine Group, Turku Centre for Biotechnology, University of Turku, Turku, Finland; 2 Department of Mathematics and Statistics, University of Turku, Turku, Finland; 3 Department of Surgery, Central Hospital of Central Ostrobothnia, Kokkola, Finland; 4 Department of Urology, Turku University Hospital, Turku, Finland; 5 Department of Pathology (HUSLAB), Helsinki University Hospital, Helsinki, Finland; 6 Institute for Molecular Medicine Finland (FIMM), University of Helsinki, Helsinki, Finland

## Abstract

Ultrasensitive prostate-specific antigen (u-PSA) remains controversial for follow-up after radical prostatectomy (RP). The aim of this study was to model PSA doubling times (PSADT) for predicting biochemical recurrence (BCR) and to capture possible discrepancies between u-PSA and traditional PSA (t-PSA) by utilizing advanced statistical modeling. 555 RP patients without neoadjuvant/adjuvant androgen deprivation from the Turku University Hospital were included in the study. BCR was defined as two consecutive PSA values >0.2 ng/mL and the PSA measurements were *log*_*2*_-transformed. One third of the data was reserved for independent validation. Models were first fitted to the post-surgery PSA measurements using cross-validation. Major trends were then captured using linear mixed-effect models and a predictive generalized linear model effectively identified early trends connected to BCR. The model generalized for BCR prediction to the validation set with ROC-AUC of 83.6% and 95.1% for the 1 and 3 year follow-up censoring, respectively. A web-based tool was developed to facilitate its use. Longitudinal trends of u-PSA did not display major discrepancies from those of t-PSA. The results support that u-PSA provides useful information for predicting BCR after RP. This can be beneficial to avoid unnecessary adjuvant treatments or to start them earlier for selected patients.

Prostate-specific antigen (PSA) is the most widely used tool to detect and monitor prostate cancer (PCa)^1^. PSA detection methods with detection levels under 0.1 ng/mL are considered ultrasensitive and some assays are capable of detecting levels approaching 0.001 ng/mL^2^. The use of ultrasensitive PSA assays (u-PSA) remains controversial due to questions regarding reliability and usefulness of u-PSA^3^. However, u-PSA could potentially detect biochemical recurrence (BCR) after radical prostatectomy (RP) significantly earlier than traditional PSA (t-PSA) assays^4^.

Early detection of BCR is important because salvage radiation therapy (RT) is most efficient when given shortly after BCR^5^. BCR is defined here as two or more consecutive PSA values over 0.2 ng/mL concordant to the EAU consensus^6^. Currently, there is no evidence that salvage RT prompted by elevated u-PSA values after RP would improve patient survival. Nevertheless, it could save high-risk patients from unnecessary adjuvant RT and favor more selective salvage RT^7^.

PSA doubling time (PSADT) has been used to estimate the risk of disease progression after radical surgery; PSADT of nine months or less is an independent risk factor for prostate cancer specific mortality^8^. Detectable u-PSA levels after RP can predict PCa recurrence^9^. Patients with undetectable u-PSA two years after surgery are unlikely to develop rapid clinical progression of PCa (PSADT <9 months if experiencing BCR later)^10^. Based on current literature, the correlation between general PSADT and ultrasensitive doubling times (u-DT) is poor^11^. False positive findings from u-PSA may also originate from laboratory measurement errors^12^,^13^.

The aim of this study was to develop novel tools that reduce the unreliability related to u-PSA. Furthermore, we assessed the potential prognostic significance of u-DT for predicting BCR after RP and applied comprehensive mathematical modeling of u-PSA and t-PSA, in order to establish an accurate predictive link between early measurements of PSA and the risk of BCR.

## Methods

### Patient material

Patients undergoing open RP and limited pelvic lymphadenectomy at Turku University Hospital during 2004–2008 were included (n = 604). The follow-up period was a minimum of 6 years. Open RP was performed as initially described by Walsh *et al*.^14^. Patients who received neoadjuvant or adjuvant androgen deprivations (ADT) were excluded; this also meant the exclusion of node positive patients, resulting in 555 patients. For practical reasons all ADTs during the follow-up period were called as adjuvant ADT, resulting population including no patients with hormonal treatment. From the 555 patients, full follow-up information was unavailable for 33 patients and 19 patients died of causes unrelated to PCa, resulting in a final set of 503 patients. Patients who died from other causes than PCa were excluded to avoid bias, as also very early follow-up information from some of these patients was lacking. Patients with adjuvant RT (ART) were not excluded based on our earlier findings demonstrating no differences between DTs in RT patients with or without adjuvant^15^. This study from Seikkula *et al*. composed of almost identical study population, and assessed optimal u-PSA threshold for upcoming BCR. In the conducted multivariate analysis there were no significant differences between patients with or without ART. According to the Finnish national rules and regulations for medical registry studies with retrospective nature no patient consents are required. Study protocol was approved by the IRB of the hospital district of South West Finland and the study was carried out taking into account all the study guidelines and national laws in Finland.

The patients were followed every 3 months for the first year after the surgery and semiannually thereafter. The follow-up included a physical examination and u-PSA measurements. Data was collected retrospectively from Turku University Hospital’s medical records and PSA data was obtained from Turku University Hospital laboratory data sources. All the PSA-analyses were done with electrochemiluminescence-immunoassay (ECLIA, Roche Diagnostics GmbH), which has a lowest limit of detection (LLD) 0.003 ng/mL. The collected data included essential clinicopathological variables, neoadjuvant and adjuvant therapies, and follow-up information, which were later used also in multivariate analysis of a potentially more accurate LASSO penalized prediction model by expanding beyond just PSA-derived information.

### Processing of PSA measurements

PSA measurements with non-detected quantities were imputed using the smallest non-zero measurement. Of all the eligible post-surgery measurements, 4502 (79.6%) were u-PSA (≤0.1 ng/mL) and 1151 (20.4%) t-PSA (>0.1 ng/mL). Post-surgery PSA nadir was defined as the lowest PSA measurement within a 3 month window after surgery. The 3 month period was chosen because 8 weeks is ample time to allow PSA levels to clear after RP and detectable u-PSA values in 1–3 months after RP are suggested as a marker for BCR progression^9^,^16^. The mathematical modeling was based only on post-nadir measurements prior to possible salvage treatments.

To evaluate the generalization ability of the modeling, the data was randomized into 3 subgroups of subjects prior to model development, where factors such as age, BCR status, and Gleason score (GS) were balanced. 2 of the subgroups were randomly chosen as the exploratory data and fully utilized in model development. Within this exploratory data, generalization ability was maintained through cross-validation. The remaining third of the data was utilized as a validation set, to retain an objective view to the robustness of the final model (Table 1).

### Mathematical modeling

Cubic penalized splines were used in the exploratory set with a wide range of values for the spline smoothing parameter λ. The optimal smoothing parameter was identified by minimizing the cross-validation Median Squared Error (MSE) of the spline fits. Penalized splines provided a flexible approach to explore whether the *log*_*2*_-transformed PSA would display complex non-linear patterns (low λ) or linear patterns (high λ).

Based on the observed highly linear patterns of the *log*_2_-transformed PSA, a linear mixed-effects model was built. The parameter estimates of the model for the *log*_2_-PSA nadir and PSADT were used for detecting differences between the BCR and non-BCR patients. A clinical risk assessment tool was further derived using generalized linear mixed-effects models as a binary classifier for BCR using parameter derivatives from the patient-wise nadir and PSADT. Furthermore, we then subjected the binary classification task of nadir and PSADT along with clinical parameters from Table 1 to penalized LASSO regression, where the multivariate regression model is optimized to maximal generalizability by penalizing the inclusion of non-zero coefficients, i.e. non-informative, overlapping or correlated variables are eliminated.

The mathematical modeling was conducted using the R statistical software (version 3.2)^17^, along with the R-packages *psplines*^18^, *lme4*^19^ and *glmnet*^20^ for the penalized cubic splines, linear mixed-effects models and penalized LASSO regression, respectively. See the Supplementary Methods for a more detailed description of the mathematical modeling process, including splines, linear mixed-effects models and the LASSO multivariate regression.

## Results

**Figure 1:**
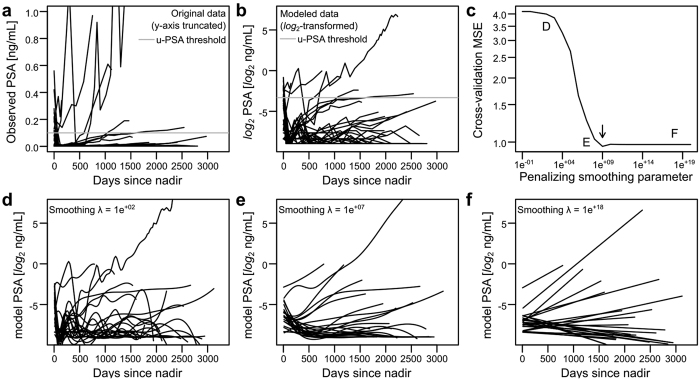
Longitudinal PSA profiles for 30 randomly chosen patients using penalized cubic splines. (**a**) The raw PSA-profiles exhibited varying patterns as a function of time since post-surgery nadir. (**b**) After *log*_2_-transformation, unit increase in the response corresponds to doubling in the original scale. (**c**) Model complexity was chosen according to Cross-Validation (CV) Median Squared Error (MSE). Optimal model (λ = 10^9^) is indicated with the arrow. (**d**–**f**) Example model fits for varying λ are shown for the *log*_2_-scale data from panel **b**.

Detailed patient characteristics are reported in Table 1. Majority of the post-surgery PSA measurements were detectable only in the u-PSA range: 83.6% and 79.1% in the exploratory and validation sets, respectively. There were 156 (46.2%) and 73 (44.2%) patients with pT3, and nearly half of the patients had Gleason ≤ 6. Rate of positive margins was approximately 40%. Only 15.4% and 13.3% of the patients reached BCR during follow-up. Representative longitudinal curves of 30 randomly chosen patients are shown prior and post to the *log*_2_-transformation in Fig. 1a,b, respectively. Due to the *log*_2_-transformation a unit change corresponded to PSA doubling in the original PSA scale. For detailed modeling results, see the Supplementary Results within the Supplementary Material.

### Splines and linear parametric models

**Figure 2:**
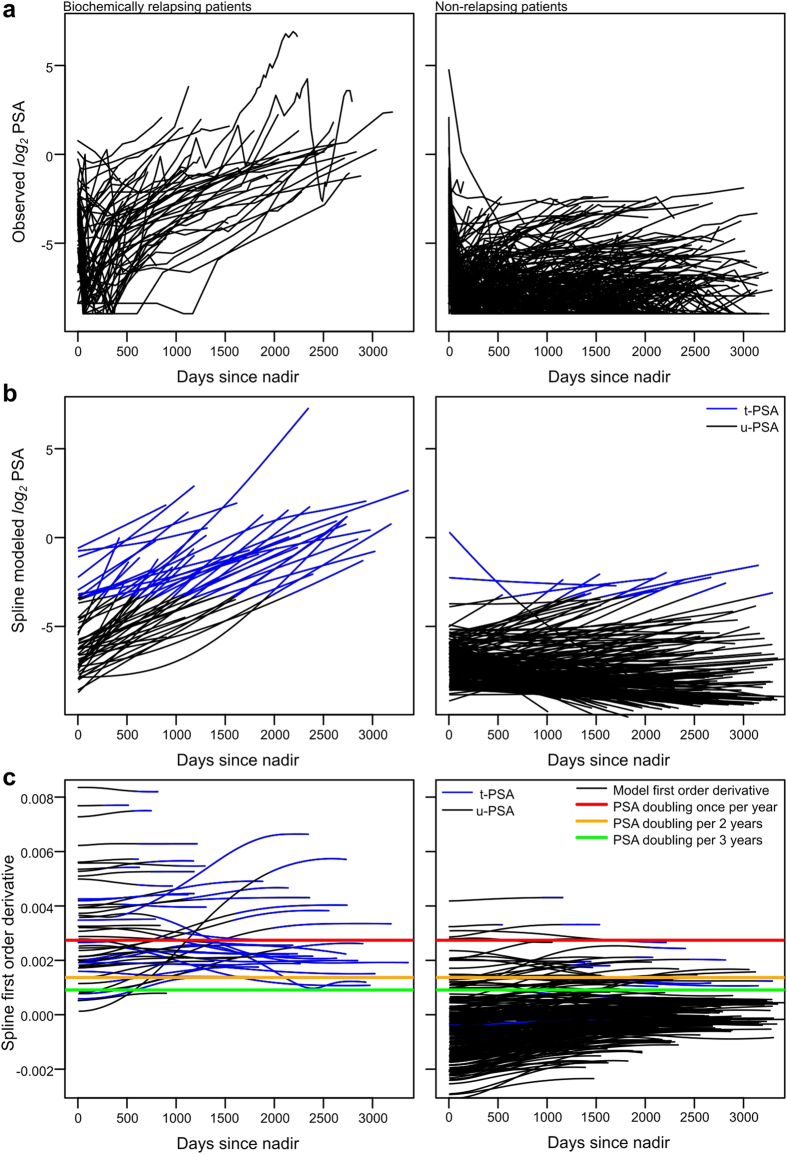
All the modeled exploratory data, model fits and the first order derivatives of the penalized splines for the relapsing (left column; *N* = 52) and non-relapsing patients (right column; *N* = 279). (**a**) Modeled *log*_2_-transformed data. (**b**) Corresponding penalized cubic spline fits. (**c**) The first order derivatives. With few exceptions, derivatives maintained relatively constant levels over the follow-up period. Once per year or once per two years PSA doubling criteria were good indicators of relapse or non-relapse of patients. Noticeable differences between u-PSA (black) and t-PSA (blue) were not present.

The major PSA trends were effectively captured readily by linear components in the model based on the optimality of high values of the smoothing parameter λ (Fig. 1c) as well as upon visual inspection (Fig. 1d–f; Fig. 2a,b). Interestingly, the first order derivatives that capture longitudinal changes in PSADT clearly distinguished between the BCR and non-BCR, suggesting that longitudinal follow-up of PSADT could provide an accurate predictor of BCR (Fig. 2c). The u-PSA and t-PSA did not exhibit markedly different patterns in the splines (Fig. 2b,c).

**Figure 3:**
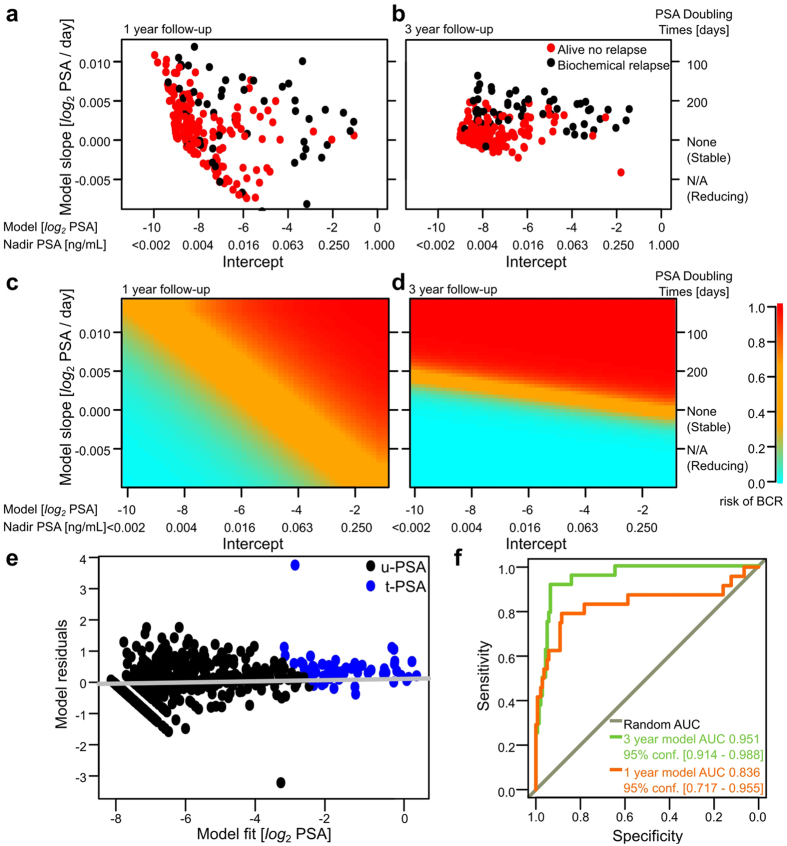
(**a**,**b**) Linear mixed-effects models yielded estimates for patient-specific nadir intercept and doubling coefficient using a 1 year (panel **a**) or a 3 year post-nadir censoring window (panel **b**). (**c**,**d**) Using generalized regression, we identified prediction surfaces for the risk of BCR using the 1 year (panel **c**) or 3 year post-nadir time window (panel **d**). Logistic regression predictions for the generalized linear models for the generalized linear models were annotated using the color key on the right. (**e**) Regression residuals for the 1 year post-nadir window using linear-mixed effects models display slight decrease in residual variance as a function of u-PSA versus t-PSA, though no systematic increasing or decreasing trends were detected. (**f**) The validation dataset suggested high predictive accuracy for BCR using the fitted models from the exploratory portion of data.

Since splines suggested that linear model families were suitable for modeling the *log*_2_-PSA patterns, we fitted linear regression models to perform parametric inference for the population effects. The focus was on the *log*_*2*_-PSA nadir and PSADT. Patient-wise estimates for these coefficients are shown in Fig. 3a,b with 1 or 3 year follow-up, respectively.

Finally, generalized linear models were used as binary classifiers to connect the patient-wise characteristics from Fig. 3a,b to the known BCR statuses. The prediction accuracy using 1 year or 3 year post-nadir follow-up was 85.3% or 88.8%, respectively, using the prediction surfaces provided in Fig. 3c,d. Overall, only minor variation was detected between the u-PSA and t-PSA in model diagnostics, exemplified by the slight decrease of heteroscedasticity over the threshold for model residuals (Fig. 3e). A computational example for predicting future patient risks with our given model estimates is provided for the mathematically inclined readers through the conventional theoretical connection to simple linear regression in the Supplementary Table S1, which generalizes to any standard spreadsheet software.

Clinical parameters, such as pT-classification or GS, in connection to the patient-wise estimates of PSA nadir and DT are reported in Supplementary Table S2. GS classes (≤6, 7 or ≥8), positive subsequent salvage treatment status, and a pre-surgery PSA >10 ng/mL were associated to differences in post-nadir PSADT estimated by the model. For the *log*_2_-PSA nadir model parameter, multiple associations were identified, excluding GS, indicating that multiple clinical parameters may be associated with the sensitive detections possible only in the u-PSA range (Supplementary Table S2). Their interpretation remains to be further studied, thus the prediction model was based solely on PSA trends.

When LASSO regression was cross-validated (CV) and the optimal model was fitted using penalization parameter within a single standard error of minimal CV error (Supplementary Fig. S2a), the multivariate regression model proposed utilizing only the estimated PSA nadir and PSADT as variables for BCR prediction. While multiple clinical variables were informative and almost included (Supplementary Fig. 2b), the generalized multivariate model highlights usefulness of the nadir and PSADT over conventional clinical parameters.

### Validation

One representative third of the data was left for objective validation of the modeling procedure in a wider context (Table 1 right panel). The validation predictions resulted in high sensitivity and specificity both for the 1 and 3 year models (Fig. 3f) with the Area Under the ROC-curve (ROC-AUC) of 0.836 (95% CI 0.72–0.96) and 0.951 (95% CI 0.91–0.99), respectively.

### Graphical user-interface pipeline for future predictions

**Figure 4:**
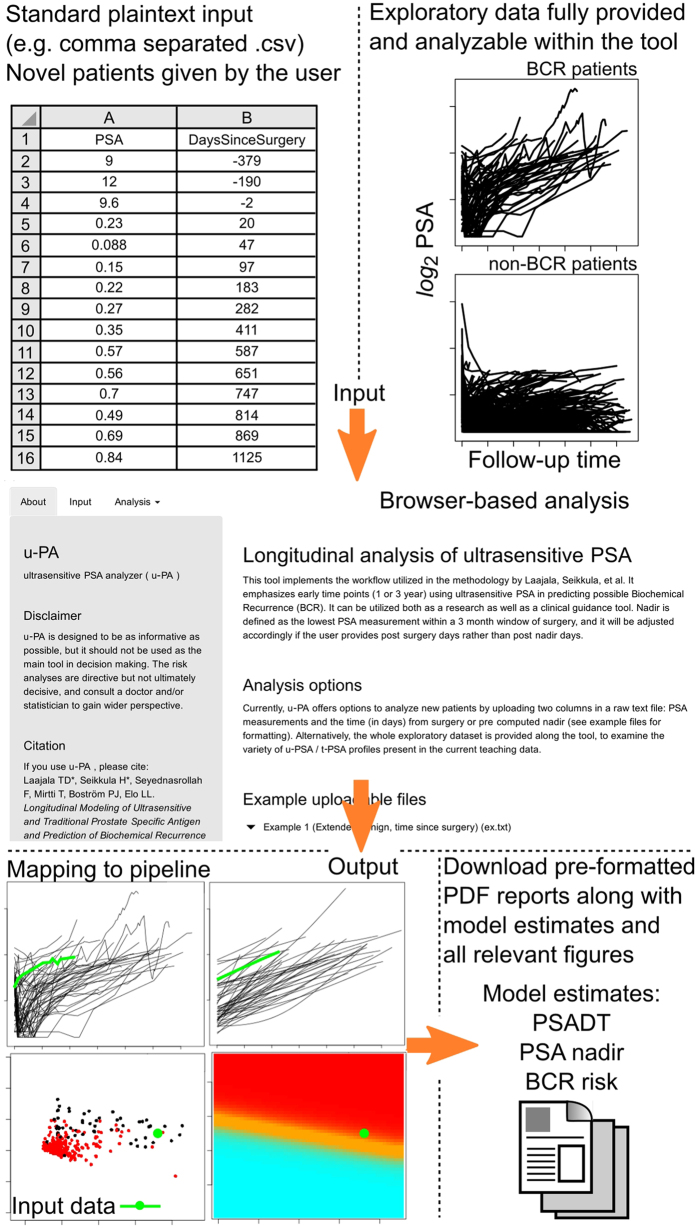
Graphical user interface workflow for predicting future patients or for analyzing the provided exploratory dataset of the current study.

In order to provide the analysis pipeline widely accessible to clinicians, a graphical user interface (GUI) was implemented using the R Shiny (RStudio Inc) platform with the underlying mathematical methodology outlined in the Supplementary Methods. The GUI is freely available at the Shinyapps.io (RStudio Inc) service-platform (http://compbiomed.shinyapps.io/u-pa/). The tool allows automated analysis of novel measurements with the existing methodology, and is provided with the exploratory dataset for illustrative purposes. Its design allows clinicians to conveniently run the pipeline and generate PDF-based risk reports for new patients. A typical workflow of the GUI is presented in Fig. 4.

## Discussion

In the current study we applied mathematical modeling to investigate the role of u-PSA as means of follow-up after RP. Based on our results, u-PSA provides useful information for predicting BCR after RP and we developed an easily applicable prediction platform (Fig. 4), which to our knowledge is the first clinically relevant predictive tool focused on u-PSA. Our results show highly linear trends in PSADT (Fig. 1). This offers a clinically convenient analysis approach, as raw PSA measurements may be transformed to PSADT through the *log*_2_-transformation, after which the linear trends may be captured using conventional tools widely available in any statistical or spreadsheet software. According to previous studies the specificity of u-PSA is poor^7^, but in our study we show that by using sophisticated computational techniques the sensitivity and specificity are high.

Based on our analysis of the second order spline derivatives, major trends in u-PSA curvature were established by the end of the first year and only slight individual variation occurred thereafter (Supplementary Fig. 1). Stabilization in the curvature of the splines suggested consistent changes in *log*_*2*_-PSA after the first follow-up year. Motivated by the spline analysis supporting linear trends (high optimal smoothing parameter λ), we tested parametric linear inference using both a 1 year and a 3 year post-nadir window. A 3 year time window was also utilized by Malik *et al*., in a study where they assessed non-detectable vs. detectable u-PSA with the threshold of 0.05 ng/mL^21^. Detectable u-PSA 2–2.5 years after RP was independent prognostic factor for PSA progression also according to Chang *et al.,* but in their study the presence of a detectable u-PSA level earlier than 2 years from surgery did not reliably predict the subsequent clinical course of BCR^11^. Although our model can predict BCR accurately already within an early follow-up window after surgery, it may still suffer from over-diagnosis related issues and patient-specific risk evaluation is recommended^12^,^22^. Earlier studies assessing the risk of PCa-progression from u-PSA have detected one year average lead time from detectable u-PSA threshold to BCR^7^. According to our analyses, the 2 year follow-up period utilized by Chang *et al*. and 3 year utilized by Malik *et al*. may already be well established by the end of the first year post-nadir^10^,^21^. In our analyses the ability to distinguish between the non-BCR (> 80%) and BCR (<20%) patients was only marginally improved if 3 years of post-nadir follow-up was allowed instead of 1 year (Fig. 3f).

Generalized linear regression model can be used as a binary classifier to evaluate BCR risks for future patients, for example by mapping the potential patients to the prediction surfaces provided by the current modeling process (Fig. 3c,d). The validation dataset in this study highly supported the hypothesis that predictive accuracy could be obtained readily by the end of the first follow-up year (Fig. 3f). We provide a practical computational example for the validated generalized linear models for predicting the BCR risk of a patient (Supplementary Table S1) and an easy-to-use graphical user interface that is freely available at http://compbiomed.shinyapps.io/u-pa/ (Fig. 4).

When t-PSA levels are used, nadir after surgery is usually undetectable (<0.1 ng/mL)^23^. In u-PSA range the undetectable level has a wider spectrum^24^. Currently the significant threshold level of u-PSA relapse is unknown. Recently we suggested a threshold between 0.03–0.05 ng/mL^15^. Malik *et al*. showed a clear association for delayed BCR with u-PSA values of <0.05 to >0.05 ng/mL 3 years after RP^21^. Previously, clear survival benefit was shown among men with low u-PSA nadir after RP^9^. In this study, the nadir intercept and PSADT estimates were found to be highly statistically significantly associated with BCR. Our definition of PSA nadir was the lowest PSA measurement within a 3 month window from RP. More sophisticated parametric methods to determine nadir include piece-wise change-point models, which can incorporate knots that are inter-connected with linear curves^25^. In order to assess the true cutoff-point for reliable u-PSAs LLD, modeling the exact time to nadir would be an interesting future research question.

In our exploratory dataset, the median time between two subsequent post-surgery PSA measurements was 152 days. Therefore, the first year of follow-up mostly consisted of 3 measurements. This amount is the minimal number of observations required to fit a linear regression model. The 3 year follow-up period was less sensitive to the nadir point and more likely holds a more realistic amount of observations for reliable PSADT estimation. However, it remains to be validated to what extent the doubling trends are established by the end of the first year, and for this purpose more intensive coverage of PSA trends would be already required for the early follow-up.

Accurate methods to determine the clinical risk represented by a rising PSA value are critical to develop rational treatment strategies. So far no studies have demonstrated that u-PSA triggered therapy will improve outcome. On the other hand, u-PSA kinetics may provide predictive information. Only few studies have compared DTs in traditional and ultrasensitive ranges^10^,^11^,^15^,^26^. It is possible that past negative findings for u-PSA have been susceptible to utilizing single measurements as predictors^10^,^11^,^26^. When multiple measurements are not considered, variation in single measurements may dominate instead of averaging more coherent trends through regression curves. This highlights the need for feasibly chosen mathematical models that capture all patient-specific variation in a more effective manner. Some authors claim that u-PSA measurements are helpful to determinate early BCR after RP^4^,^27^,^28^. Others claim that it will offer no benefit and mainly cause unnecessary anxiety for patients^29^. Previously Reese *et al*. demonstrated a poor correlation between PSADTs, possibly due to inconsistency of u-PSA measurements^11^. Some authors have reported unreliability of u-PSA measurements^13^,^30^. Also according to literature, specificity of the u-PSA is relatively poor^7^. In our study, when utilizing sophisticated mathematical modeling over time we identified no major discrepancies between the u-PSA and t-PSA. In contrast, large portion of our data at 1 year window post-nadir consisted of u-PSA (Table 1; median 3 measurements by the end of the first follow-up year), while retaining a good prediction and generalization ability. Most importantly, because u-PSA may improve the time to detection as a supplement to t-PSA of BCR by months or years, this advantage of earlier prediction for relapse has potential to improve the patients’ chance of durable progression-free survival with salvage therapy given at a lower cancer burden and a wider time window for cure^4^,^31^. Furthermore, by presenting mathematically extensive approach with both univariate and multivariate modeling of BCR, we highlight the need of accurate prediction tools that outperform and raise awareness that arbitrary chosen simple thresholds (e.g. in t-PSA range) are likely to be subpar.

Major strength of this study is the extensive mathematical modeling of both the u-PSA and t-PSA measurements, all of which is offered as an easy to use web-based graphical user interface (GUI) platform. All the PSA measurements were done with the same PSA assay, reducing error caused by varying assays. A limitation of the study is that all the patients were from the same hospital district, and thus a larger sample size and longer follow-up is needed for more accurate validation of these findings in order to guarantee generalizability.

## Conclusions

Our results indicate that u-PSA provides useful information for predicting BCR after RP. The utilized approach of considering PSADT was easily achieved using *log_2_*-transformation of the data, and makes our conclusions and estimates comparable to any study utilizing the well-established PSADT as an end-point^32^,^33^. Using this convenient approach, we developed a novel mathematical modeling a mathematical modeling pipeline and implementation utilizing only PSADT and PSA nadir for predicting BCR mainly based on u-PSA measurements in early follow-up of PSA response. To our knowledge this is the first clinically relevant predictive tool focused on systematically complementing t-PSA with u-PSA and displaying the coherence between the two; however, for future studies to be clinically widely applicable, albeit thresholds have been suggested^15^, a more extensive exploration of u-PSA key thresholds is imperative. We believe that such threshold most likely would be a combination of the estimated nadir (in u-PSA range), the readily established PSADT potentially expanding both u-PSA and t-PSA ranges, and would most likely also include other clinically relevant variables (Supplementary Table S2). We believe that in salvage RT policy early risk evaluation is beneficial and we are optimistic about the predictive use of u-PSA in supplement to the more established t-PSA measurements. Our easily accessible mathematical pipeline established a novel baseline for future validation studies of u-PSA importance and method development.

## Additional Information

**How to cite this article**: Laajala, T. D. *et al*. Longitudinal modeling of ultrasensitive and traditional prostate-specific antigen and prediction of biochemical recurrence after radical prostatectomy. *Sci. Rep.* **6**, 36161; doi: 10.1038/srep36161 (2016).

**Publisher’s note:** Springer Nature remains neutral with regard to jurisdictional claims in published maps and institutional affiliations.

## Supplementary Material

Supplementary Information

## Figures and Tables

**Table 1:** Patient characteristics, PSA measurement counts, and patient counts in the exploratory and validation datasets (proportions in parentheses).

*Variable*	*Instance*	Dataset			
		*Exploratory 2/3*		*Validation 1/3*	
**pT**	**2**	180 (53.3%)		92 (55.8%)	
	**3**	156 (46.2%)		73 (44.2%)	
	**4**	1 (0.3%)			
	**Missing**	1 (0.3%)			
**Gleason score (GS)**	**≤6**	157 (46.4%)		80 (49.1%)	
	**7 (3** **+** **4)**	101 (29.9%)		49 (30.1%)	
	**7 (4** + **3)**	50 (14.8%)		18 (11.0%)	
	**≥8**	28 (8.3%)		16 (9.8%)	
	**Missing**	2 (0.6%)			
**Margins**	**Negative**	200 (59.2%)		100 (60.6%)	
	**Positive**	137 (40.5%)		65 (39.4%)	
	**Missing**	1 (0.3%)			
**Adjuvant RT**	**No**	295 (87.3%)		147 (89.1%)	
	**Yes**	42 (12.4%)		18 (10.9%)	
	**Missing**	1 (0.3%)			
**Salvage RT**	**No**	275 (81.4%)		136 (82.4%)	
	**Yes**	63 (18.6%)		29 (17.6%)	
**PSA at surgery**	**<10**	251 (74.3%)		121 (73.3%)	
	**10–20**	67 (19.8%)		36 (21.8%)	
	**≥20**	19 (5.6%)		8 (4.8%)	
	**Missing**	1 (0.3%)			
**Age**	**<60**	123 (36.4%)		61 (37.0%)	
	**60–70**	193 (57.1%)		96 (58.2%)	
	**>70**	21 (6.2%)		8 (4.8%)	
	**Missing**	1 (0.3%)			
**Total counts of PSA measurements in different time windows**	**Time post-surgery**	*t-PSA*	*u-PSA*	*t-PSA*	*u-PSA*
	**<1y**	166	875	161	466
	**1y–3y**	120	788	78	413
	**>3y**	236	1000	164	649
**Patient status**	**No recurrence**	279 (82.5%)		140 (84.8%)	
	**Recurrence (BCR)**	52 (15.4%)		22 (13.3%)	
	**Metastasis/other**	7 (2.1%)		3 (1.8%)	
