# Erratum: Longitudinal modeling of ultrasensitive and traditional prostate-specific antigen and prediction of biochemical recurrence after radical prostatectomy

**DOI:** 10.1038/srep40835

**Published:** 2017-01-19

**Authors:** Teemu D. Laajala, Heikki Seikkula, Fatemeh Seyednasrollah, Tuomas Mirtti, Peter J. Boström, Laura L. Elo

Scientific Reports 6: Article number: 36161; 10.1038/srep36161 published online: 11 02 2016; updated: 01 19 2017.

The original version of this Article contained an error in the spelling of Fatemeh Seyednasrollah, which was incorrectly given as Fatemeh Seyednasrollah sadat.

This has now been corrected in the HTML and PDF versions of this Article.

