# Supplementary material for: Longitudinal modeling of ultrasensitive and traditional prostate-specific antigen and prediction of biochemical recurrence after radical prostatectomy
Source: Sci Rep. 2016 Nov 2;6:36161. doi: 10.1038/srep36161 (PMC5090356; doi:10.1038/srep36161)
Supplement: Supplementary Information [file srep36161-s1.pdf]

Supplementary Document for

Longitudinal modeling of ultrasensitive and traditional prostate-specific antigen and prediction of biochemical recurrence after radical prostatectomy

Teemu D. Laajala [1], Heikki Seikkula [1], Fatemehsadat Seyednasrollah, Tuomas Mirtti, Peter J. Boström, Laura L. Elo.

1: Equal contribution

**Supplementary Figure S1.**	Second order derivatives of the spline fits. (**a**) Biochemically relapsing patients (BCR, N=52), (**b**) non-BCR patients (N=279). The vertical light and dark grey lines indicate one year and three year time points, respectively. Corresponding fitted measurements for the u-PSA and t-PSA are annotated using black and red colors, respectively.
**Supplementary Figure S2.**	LASSO model cross-validation and penalization curve. (**a**) 10-fold cross-validation (CV), based on which optimal penalization was chosen to be first penalization parameter within a standard error of the CV minimum. (**b**) Penalization curves, which display that the log_2 nadir and PSADT were selected by the final model, albeit some traditional clinical parameters were almost included.
**Supplementary Table S1.**	A computational spreadsheet example of simple linear regression in predicting BCR risk from the proposed generalized linear model.
**Supplementary Table S2.**	Estimated 3 year follow-up patient-wise log2-PSA nadir levels (intercepts) and PSADT (doubling slopes) in the exploratory dataset in connection to the patients' clinical parameters.
**Supplementary Methods and Results.**	Extended mathematical methodology as well as more detailed results of the utilized regression methods.

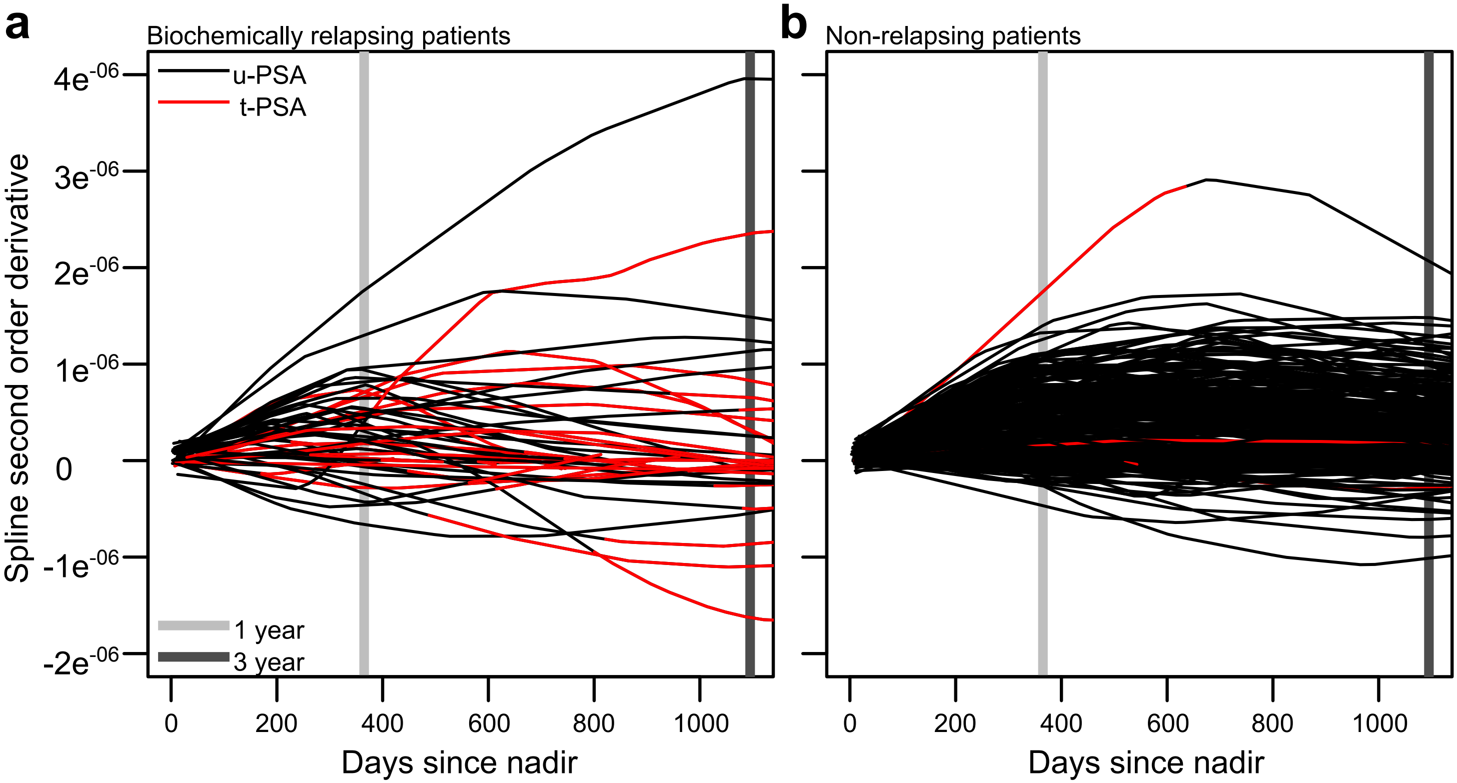

a
Biochemically relapsing patients
u-PSA
t-PSA
1 year
3 year
Spline second order derivative
4e-06
3e-06
2e-06
1e-06
0
-1e-06
-2e-06
0
200
400
600
800
1000
Days since nadir
b
Non-relapsing patients
Days since nadir

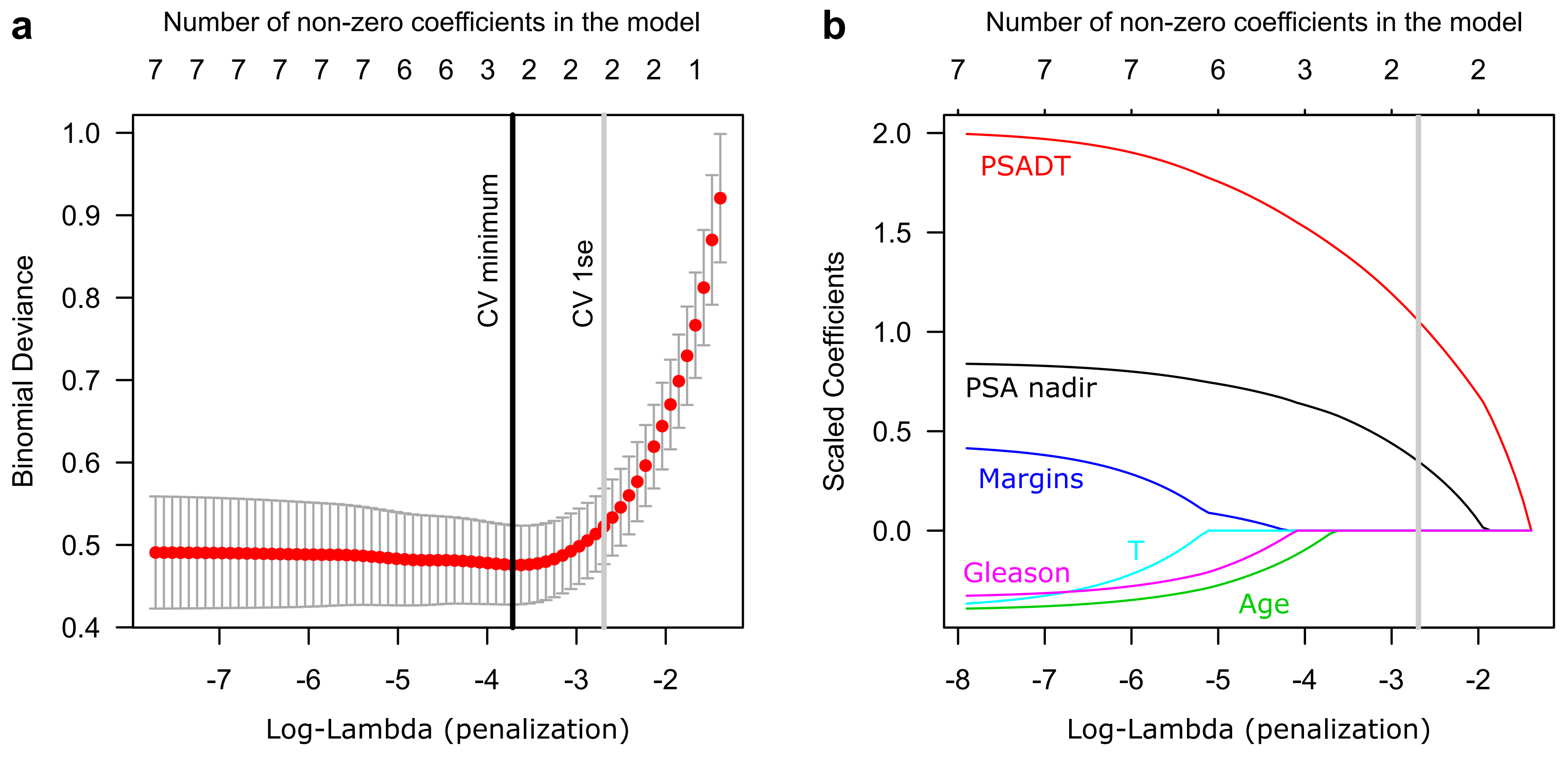
a
Number of non-zero coefficients in the model
7 7 7 7 7 7 6 6 3 2 2 2 2 1
Binomial Deviance
1.0
0.9
0.8
0.7
0.6
0.5
0.4
CV minimum
CV 1se
-7 -6 -5 -4 -3 -2
Log-Lambda (penalization)
b
Number of non-zero coefficients in the model
7 7 7 6 3 2 2
Scaled Coefficients
2.0
1.5
1.0
0.5
0.0
PSADT
PSA nadir
Margins
T
Gleason
Age
-8 -7 -6 -5 -4 -3 -2
Log-Lambda (penalization)

Supplementary Table S1: A computational spreadsheet example of simple linear regression in predicting BCR risk from the proposed generalized linear model

Col → Row ↓	**A** **PSA**	**B** ***log*₂-PSA (= y)**	**C** **DaysSinceSurgery**	**D** **DaysSinceNadir (= x)**	**E** x^2	**F** $x \cdot y$
**1**	10	3.321928	-139	-184		
**2**	7.6	2.925999	-1	-46		
**3**	0.091	-3.45799	21	-24		
**4**	0.006	-7.381	45	0	0	0
**5**	0.022	-5.506	78	33	1089	-181.7
**6**	0.033	-4.921	100	55	3025	-270.7
**7**	0.006	-7.381	225	180	32400	-1329
**8**	0.004	-7.966	335	290	84100	-2310
**9**	0.003	-8.381	710	665		
**10**	0.003	-8.381	1092	1047		
**11**	0.003	-8.381	1289	1244		
**12**	0.008	-6.966	1429	1384		
**13**	0.006	-7.381	1584	1539		

Each row corresponds to a single PSA measurement. In our current study, we defined nadir to be the lowest point in PSA within a 3-month window post-surgery, thus limiting observations for our model to observations $Row \geq 4$ in this example. Similarly, in order to evaluate model parameters in a 1-year window post-nadir, the lower limit for utilized observations is set at $Row \leq 8$. These constraints were obtained by observing the days since nadir column at **D**. The simple regression coefficients can be computed in closed form:

$$\begin{aligned}
\overline{x^2} &= \text{AVERAGE(E4:E8)} &&= 24123 \\
\overline{xy} &= \text{AVERAGE(F4:F8)} &&= \text{-}818.2 \\
\bar{x} &= \text{AVERAGE(D4:D8)} &&= 111.6 \\
\bar{y} &= \text{AVERAGE(B4:B8)} &&= \text{-}6.63103 \\
\bar{x}^2 &= \text{POWER(AVERAGE(D4:D8), 2)} &&= 111.6^2 = 12454.56
\end{aligned}$$

Thus, for this particular individual, the simple regression estimates are:

$$\begin{aligned}
\hat{\beta_2} &= \frac{\overline{xy} - \bar{x}\bar{y}}{\overline{x^2} - \bar{x}^2} &&= \frac{-818.2 - (111.6 \cdot -6.63103)}{24123 - 12454.56} &&= -0.00669987 && \text{(PSADT)} \\
\hat{\beta_1} &= \bar{y} - \hat{\beta_1}\bar{x} &&= -6.63103 - (-0.00669987 \cdot 111.6) &&= -5.883325 && (log_2\text{-PSA nadir})
\end{aligned}$$

where $\hat{\beta_2}$ corresponds to the PSADT and $\hat{\beta_1}$ to the log_2-PSA nadir. Above estimates may be inspected in **Figure 3 D** to evaluate the individual's risk for BCR. In this particular case, the risk for BCR is very low, which is expected when the PSADT coefficient is negative (no doubling occurs). 1 year follow-up was used as a criterion for including observations in estimating $\hat{\beta_1}$ and $\hat{\beta_1}$. The coefficients $\{\beta_{base}$, β_{nadir} and $\beta_{doubling}\}$ reported in our study for 1-year follow up were $\{2.736, 0.640, 218.488\}$. Thus, the risk for BCR for this individual may be computed as provided in the **Supplementary Methods**:

$$\frac{1}{1 + e^{-(\beta_{base} + \beta_{nadir} \times x_1 + \beta_{doubling} \times x_2)}} = \frac{1}{1 + e^{-(2.736 + 0.640 \cdot -5.883325 + 218.488 \cdot -0.00669987)}}$$

$$= 0.07633843...$$

which would indicate a very low risk of BCR, as was later observed in follow-up. Similarly, a BCR risk for a hypothetical patient undergoing PSADT every 150 days ($1/150 \approx 0.00667$) and an estimated log_2-PSA nadir of -5 (or $2^{-5} = 0.03125$ in the original PSA scale) would yield ≥ 0.5 risk:

$$\frac{1}{1 + e^{-(2.736 + 0.640 \cdot -5 + 218.488 \cdot 0.00667)}} = 0.7297422...$$

Supplementary Table S2: Estimated 3 year follow-up patient-wise *log*$_2$-PSA nadir levels (intercepts) and PSADT (doubling slopes) in the exploratory dataset in connection to the patients' clinical parameters

		log$_2$-PSA nadir (intercepts $\beta_0 + \gamma_{0,i}$)						PSADT (slopes $\beta_1 + \gamma_{1,i}$)						
		Min.	***1st Qu.***	***Median***	***Mean***	***3rd Qu.***	***Max.***	***Min.***	***1st Qu.***	***Median***	***Mean***	***3rd Qu.***	***Max.***	***N***
**pT**	**2**	-10.448	-8.542	-8.266	-7.877	-7.729	-2.557	-0.007475	-0.000039	0.000322	0.000804	0.000797	0.006146	*179*
	**3**	-9.603	-8.374	-7.815	-7.131	-6.045	-1.432	-0.001640	-0.000058	0.000474	0.001190	0.002202	0.006671	*154*
**Gleason score**	**<=6**	-9.115	-8.588	-8.065	-7.904	-7.699	-5.418	-0.000488	-0.000064	0.000195	0.000558	0.000569	0.005971	*59*
	**7**	-9.100	-8.620	-7.990	-7.488	-7.031	-2.568	-0.000371	-0.000044	0.000297	0.001097	0.002097	0.006633	*58*
	**>=8**	-10.448	-8.903	-7.864	-7.791	-7.216	-3.815	-0.000565	0.000384	0.002190	0.002002	0.003372	0.005052	*12*
**Margins**	**Neg.**	-10.448	-8.526	-8.226	-7.772	-7.633	-1.432	-0.001640	-0.000062	0.000322	0.000858	0.000853	0.006671	*199*
	**Pos.**	-9.115	-8.405	-7.815	-7.176	-6.045	-1.651	-0.000551	-0.000049	0.000483	0.001169	0.002148	0.006633	*134*
**Adjuvant RT**	**No**	-10.448	-8.494	-8.161	-7.656	-7.408	-1.432	-0.001640	-0.000044	0.000345	0.000947	0.001137	0.006671	*293*
	**Yes**	-9.112	-8.386	-7.010	-6.627	-5.352	-1.651	-0.000551	-0.000056	0.000356	0.001246	0.002266	0.005044	*40*
**Salvage RT**	**No**	-9.282	-8.537	-8.236	-7.885	-7.650	-2.852	-0.001640	-0.000118	0.000194	0.000425	0.000586	0.005677	*273*
	**Yes**	-10.448	-7.901	-5.916	-5.929	-4.246	-1.432	0.000072	0.002534	0.003813	0.003519	0.004555	0.006671	*60*
**PSA at surgery**	**<10**	-10.448	-8.531	-8.177	-7.738	-7.506	-2.557	-0.000748	-0.000096	0.000276	0.000829	0.000929	0.006671	*248*
	**10-20**	-9.282	-8.409	-8.065	-7.104	-6.128	-1.432	-0.000342	0.000201	0.000704	0.001412	0.002175	0.005971	*67*
	**>20**	-9.603	-8.304	-7.273	-6.288	-4.362	-1.651	-0.001640	-0.000015	0.000506	0.001503	0.003216	0.004756	*18*

Holm-method multiple testing corrected *p*-values according to one-way ANOVA: white N.S.; orange $p<0.05$.

Longitudinal modeling of ultrasensitive and traditional prostate-specific antigen and prediction of biochemical recurrence after radical prostatectomy

Teemu D. Laajala *, Heikki Seikkula *,
Fatemehsadat Seyednasrollah , Tuomas Mirtti ,
Peter J. Boström , Laura L. Elo

Supplementary Material

Supplementary Methods

1 Cubic smoothing splines

The log_2-transformed PSA measurements y at the indicated post-nadir time points x were modeled using natural cubic smoothing splines of the generalized form:

$$f(x) = \begin{cases} A_0x^3 + B_0x^2 + C_0x + D_0 & , t_0 \leq x \leq t_1 \\ ... \\ A_{n-1}x^3 + B_{n-1}x^2 + C_{n-1}x + D_{n-1} & , t_{n-1} \leq x \leq t_n \end{cases} \tag{1}$$

Given the observed time interval $[a_i, b_i]$ in the data, smoothing splines are defined using intervals t with $a_i = t_0 < t_1 < ... < t_{n-1} < t_n = b_i$. Per each individual i, the identified solution minimized the target function:

$$\sum (y - f(x))^2 + \lambda \int_a^b f''(x) \tag{2}$$

In Eq. 2, the first term is the Sum of Squared Errors (SSE) and measures how well the model captured the observed PSA values y. The second term, the so-called penalization term, controls the amount of complexity in the model, as small values in this term indicate linear trends in the optimal model. The coefficient λ is a smoothing parameter that defines the ratio of the SSE and penalization terms, and it was determined using Cross-Validation (CV) in our approach. As such, the method provides a piece-wise identified polynomial function that establishes a balance between capturing observed variation in the data (term SSE in Eq. 2), while controlling for over-fitting by smoothing the function (the λ-term).

As the smoothing parameter $\lambda \to \infty$, the estimated splines converge towards least squares linear regression fit, establishing a convenient link between the flexible splines and the more traditional linear regression models [1]. The results

*Equal contribution

of CV for λ were thus used to motivate linear parametric model choices in downstream analyses.

A minimum of 5 observations were required for fitting a spline per each individual i, thus patients with less post-nadir measurements than 5 were filtered out of spline fitting. This resulted in a total of 306 suitable patients for spline fitting in the exploratory data portion. The above-defined penalized natural smoothing splines were fitted using the *pspline*-package [2] in the R statistical software [3].

2 Linear mixed-effects models

Motivated by the optimality of high λ-values in spline smoothing, we conducted further modeling of the log_2-PSA y using linear mixed-effects models [4] in the lme4 R-package [5]. In this approach, we could identify for each patient i a nadir intercept (γ_0) and a coefficient for PSADT (γ_1). Given the pairs of log_2-PSA $y_{i,t}$ at post-nadir day $x_{i,t}$ for a patient i, the model was fitted using:

$$y_{i,t} = \beta_0 + \beta_1 \times x_{i,t} + \gamma_{0,i} + \gamma_{1,i} \times x_{i,t} + \epsilon_{i,t} \tag{3}$$

The model in Eq. 3 was fitted both using a 1-year and a 3-year post-nadir window, restricting PSA measurements to only these time ranges. After this, the patient-level parameters $\gamma_{0,i}$ and $\gamma_{1,i}$ were summed to the population-estimates β_0 and β_1, respectively. These estimates correspond to the patient-wise log_2 PSA levels at nadir and the PSA post-nadir doubling coefficients. Notice that the regression models were agnostic in respect to the biochemical relapse statuses up to this point. Statistical significance of the fixed effects in the models were computed using the Satterthwaite approximation in the R-package *lmerTest* [6].

3 Generalized linear regression

In order to couple the extracted individual-level features ($\{\beta_0 + \gamma_{0,i}\}$ and $\{\beta_1 + \gamma_{1,i}\}$ for each patient i) with the binary biochemical relapse statuses $relapse_i \in \{0, 1\}$, generalized linear regression was utilized with the *logit*-link function. The generalized linear regression models were used to model the chance of biochemical relapse as a function of patient-wise PSA trends, which had been estimated using the linear mixed-effects models as described previously.

Let $p(x)$ denote the probability of observing a biochemical relapse for a given patient. Using generalized linear regression with the *logit*-link function:

$$log\frac{p(x)}{1-p(x)} = \beta_{base} + \beta_{nadir} \times \overbrace{(\beta_0 + \gamma_{0,i})}^{x_1} + \beta_{doubling} \times \overbrace{(\beta_1 + \gamma_{1,i})}^{x_2} + \epsilon_i \tag{4}$$

Here, β_{base} describes the base chance of observing a biochemical relapse. x_1 and x_2 are upstream model parameters extracted from the linear mixed-effects model in Eq. 3 for patient-wise PSA nadir intercept (γ_0) and PSADT coefficient (γ_1). Due to diminishing effects, a unit increase in x_1 or x_2 does not

result in a linear increase in probability of biochemical relapse $p(x)$, but instead as an increase in log odds. These changes in log odds are described in the model estimates β_{nadir} and $\beta_{doubling}$ for patient-wise PSA nadir and PSADT coefficients respectively.

As the modeled responses in logit regression capture probabilities for observing a negative (no biochemical relapse) or positive (biochemical relapse) outcome, it is natural to extend the model obtained from Eq. 4 as a classifier by predicting for patients:

$$\begin{cases} \frac{1}{1+e^{-(\beta_{base}+\beta_{nadir}\times x_1+\beta_{doubling}\times x_2)}} \leq 0.5 \rightarrow \text{no BCR} \\ \frac{1}{1+e^{-(\beta_{base}+\beta_{nadir}\times x_1+\beta_{doubling}\times x_2)}} > 0.5 \rightarrow \text{BCR} \end{cases} \tag{5}$$

Using Eq. 5 we reported prediction conventional accuracies, sensitivities and specificities both in the exploratory dataset which was used to determine β, but also in the external validation data set which was withheld from model development. Furthermore, the cut-off threshold of 0.5 may be adjusted to better reflect a desired trade-off between sensitivity and specificity of the model.

4 Connection to simple linear regression

Natural extensions to the current study include such research questions as (i) to what extent the current study can be generalized; and (ii) how could one map a new patient (or patients) conveniently to the currently obtained models. These questions were carefully considered when designing the current analysis pipeline.

Firstly, in respect to question (i), there exists a convenient link between the non-linear splines and conventional linear regression; as the $\lambda \rightarrow \infty$, the spline fit to any given data converges towards a linear least squares fit to the said data [1]. In our dataset, high values of λ established more optimal model fits as seen in the cross-validation error, and therefore linear regression could serve as a useful approximation to the splines. We therefore suggest the model parameters to be proxied by linear regression models, which can easily be generalized to any other PSA study.

Secondly, the utilized linear mixed-effects models attempt to capture population-wise thinking in modeling normally distributed parameters (γ_0 and γ_1) that build on the respective fixed effects (β_0 and β_1). New observations are expected to behave according to the fixed effects β, but may be given a certain amount of uncertainty in their deviation from population means. If future patients follow the same population distributions γ, their respective values could be estimated and evaluated in the light of our currently observed data. For this purpose we propose to utilize simple linear regression for a single patient:

$$y_t = \hat{\beta}_1 + \hat{\beta}_2 \times x_t + \epsilon_t \tag{6}$$

where y_t is the log_2-transformed PSA value corresponding to the post-nadir day x_t. It is straight-forward to show [7] that the least squares estimate for model

parameters in Eq. 6 are given by (omitting index t):

$$\begin{cases} \hat{\beta_2} = \dfrac{\overline{xy} - \bar{x}\bar{y}}{\overline{x^2} - \bar{x}^2} \\ \hat{\beta_1} = \bar{y} - \hat{\beta_1}\bar{x} \end{cases} \tag{7}$$

where y are the log_2-transformed PSA values, x are the corresponding post-nadir time in days, and $\bar{x}$ indicates the mean of x. To predict future patients, the simplified $\hat{\beta_1}$ and $\hat{\beta_2}$ may be proxied as x_1 and x_2 for Eq. 5. A computational spreadsheet example for this purpose is shown in **Supplementary Table S1**, where a randomly chosen example patient is shown along with the $\hat{\beta}$ estimates from Eq. 7. Further, such a patient may be mapped in respect to the predicted risk in **Fig. 3c-d** for biochemical relapse.

5 Penalized multivariate LASSO regression

Penalized LASSO regression is a popular multivariate regression method with an embedded feature selection methodology [8]. The model fit involves two components: (i) the conventional model likelihood; (ii) A penalization coefficient λ and an additional optional parameter with $\alpha = 1$ as a special case for LASSO. Optimal λ is estimated typically using Cross-Validation (CV), while the model fitting procedure solves optimization problem in respect to the provided model coefficients β and a given sequence of λs:

$$\min_{\beta_0,\beta} \frac{1}{N} \sum_{i=1}^{N} w_i l(y_i, \beta_0 + \beta^T x_i) + \lambda \left[(1-\alpha)||\beta||_2^2/2 + \alpha||\beta||_1\right] \tag{8}$$

Binomial link function was used to bring the regression problem to a feasible range $y \in [0, 1]$.

Supplementary Results

1 Cubic smoothing splines

While the optimal point in the Cross-Validation Median Squared Error (CV-MSE) curve was obtained when $MSE(\lambda = 109) \approx 0.9545$, the obtained error values for high λ were close to this minimum error, whereas low λ produced higher CV-MSE (Figure 1 C). This highlighted the linear nature of the PSADT curves and that linear parametric models could be effectively utilized to model the data.

2 Linear mixed-effects models

The fixed effects estimates in the linear mixed-effects models (MEM) $\{\beta_0, \beta_1\}$ yielded $\{-7.687, 1.631 \times 10^{-3}\}$ and $\{-7.604, 1.188 \times 10^{-3}\}$ for the 1 year or 3 year models, respectively. The population average PSADT (β_1) estimated 613 days and 842 days, for 1 and 3 year follow-up respectively. This indicates that

PSADT in our whole patient population approximated to two to three years regardless of BCR status. The population average nadir intercept (β_0) observed at $2^{-7.6} \approx 0.005$ ng/mL (both 1 and 3 year follow-up models) was very close to the known LLD in the original scale (0.003 ng/mL). Both the nadir (β_0) and PSADT (β_1) were highly statistically significant with $p < 0.001$.

3 Generalized linear models

The individual patients were summarized for the purposes of BCR prediction by combining the MEM population effects (β) with patient-specific random effects (γ) (Figure 3 A – B). These combined patient-wise model estimates $\{\beta_0 + \gamma_{0,i}, \beta_1 + \gamma_{1,i}\}$ as shown in Figure 3 A – B were used to determine BCR prediction surfaces in Figure 3 C - D using generalized linear models (i.e. binary classifier for BCR risk). New binary classifier model parameters $\{\beta_{base}, \beta_{nadir}, \beta_{doubling}\}$ were estimated, with a base chance of BCR (β_{base}), and patient-wise log2-PSA nadir (β_{nadir}) in $\beta_0 + \gamma_{0,i}$ and PSADT ($\beta_{doubling}$) in $\beta_1 + \gamma_{1,i}$. The 1 year post-nadir follow-up period estimated $\{2.736, 0.640, 218.488\}$ with p-values $\{8.48 \times 10^{-5}, 4.06 \times 10^{-11}, 2.99 \times 10^{-6}\}$. Using the 3 year post-nadir follow-up, the model estimated $\{0.122, 0.534, 1029.734\}$ with p-values $\{0.896, 1.42 \times 10^{-5}, 3.09 \times 10^{-13}\}$. Both the patient-wise log2-PSA nadir (β_{nadir}) and PSADT ($\beta_{doubling}$) were highly statistically significantly associated to the BCR risk in both the 1 and 3 year follow-up models ($p < 0.001$).

4 Penalized LASSO model

In the estimated LASSO model, the conservative CV-penalization of CV_{1se} (**Supplementary Figure S2a**) resulted in a model where only log_2 PSA nadir and PSADT were included in the model. The other clinical parameters were relatively equally important in respect to the binomial prediction task, while PSADT clearly outperformed the nadir, and nadir outperformed the conventional clinical parameters (**Supplementary Figure S2b**).

References

1. Ramsay, J.O., Heckman, N., & Silverman, B.W. Spline smoothing with model-based penalties. *Behav Res Methods Instrum Comput* **29**: 99-106 (1997).

2. Ripley, B. (2013). *pspline*: Penalized Smoothing Splines. Smoothing splines with penalties on order m derivatives (R-package version 1.0-16). URL http://CRAN.R-project.org/package=pspline

3. R Development Core Team (2015). *R*: a language and environment for statistical computing (version 3.2.2). R Foundation for Statistical Computing, Vienna, Austria. URL http://www.R-project.org

4. Gelman, A., & Hill, J. Data analysis using regression and multilevel/hierarchical models (eds. Alvarez R.M. et al.; Cambridge University Press, 2007). ISBN 9780521686891

5. Bates, D.M., Maechler, M., Bolker, B. & Walker, S. (2014). *lme4*: Linear mixed-effects models using S4 classes (R-package version 1.1-7). URL http://CRAN.R-project.org/package=lme4

6. Kuznetsova, A. (2014). *lmerTest*: Tests for random and fixed effects for linear mixed effect models (R-package version 2.0-6). URL http://CRAN.R-project.org/package=lmerTest

7. Weisberg, S. Applied Linear Regression (Wiley Series in Probability and Statistics, Third edition; 2005). ISBN 0-471-66379-4

8. Friedman, J.H., Hastie, T., Tibshirani, R. Regularization Paths for Generalized Linear Models via Coordinate Descent. *J Stat Soft* **33**:1-22 (2010).
